# Predicting conversion to psychosis using machine learning: response to Cannon

**DOI:** 10.3389/fpsyt.2024.1520173

**Published:** 2025-01-15

**Authors:** Jason Smucny, Tyrone D. Cannon, Carrie E. Bearden, Jean Addington, Kristen S. Cadenhead, Barbara A. Cornblatt, Matcheri Keshavan, Daniel H. Mathalon, Diana O. Perkins, William Stone, Elaine F. Walker, Scott W. Woods, Ian Davidson, Cameron S. Carter

**Affiliations:** ^1^ Department of Psychiatry, University of California, Davis, Davis, CA, United States; ^2^ Department of Psychology, Yale University, New Haven, CT, United States; ^3^ Department of Psychiatry, Yale University, New Haven, CT, United States; ^4^ Department of Psychiatry, Semel Institute for Neuroscience and Human Behavior, University of California, Los Angeles, Los Angeles, CA, United States; ^5^ Biobehavioral Sciences and Psychology, Semel Institute for Neuroscience and Human Behavior, University of California, Los Angeles, Los Angeles, CA, United States; ^6^ Department of Psychiatry, University of Calgary, Calgary, AB, Canada; ^7^ Department of Psychiatry, University of North Carolina Chapel Hill, Chapel Hill, NC, United States; ^8^ Department of Psychiatry Research, Zucker Hillside Hospital, New York, NY, United States; ^9^ Department of Psychiatry, Harvard University, Cambridge, MA, United States; ^10^ Department of Psychiatry, University of California, San Francisco, San Francisco, CA, United States; ^11^ Department of Psychiatry, University of San Diego, San Diego, CA, United States; ^12^ Department of Psychiatry, Emory University, Atlanta, GA, United States; ^13^ Department of Computer Science, University of California, Davis, Davis, CA, United States; ^14^ Department of Psychiatry, University of California, Irvine, Irvine, CA, United States

**Keywords:** schizophrenia, clinical high risk (CHR), NAPLS, out of sample evaluation, scale of psychosis risk symptoms, generalizability

## Abstract

**Background:**

We previously reported that machine learning could be used to predict conversion to psychosis in individuals at clinical high risk (CHR) for psychosis with up to 90% accuracy using the North American Prodrome Longitudinal Study-3 (NAPLS-3) dataset. A definitive test of our predictive model that was trained on the NAPLS-3 data, however, requires further support through implementation in an independent dataset. In this report we tested for model generalization using the previous iteration of NAPLS-3, the NAPLS-2, using the identical machine learning algorithms employed in our previous study.

**Method:**

Standard machine learning algorithms were trained to predict conversion to psychosis in clinical high risk individuals on the NAPLS-3 dataset and tested on the NAPLS-2 dataset.

**Results:**

NAPLS-2 and -3 individuals significantly differed on most features used in machine learning models. All models performed above chance, with Naive Bayes and random forest methods showing the best overall performance. Importantly, however, overall performance did not match those previously observed when using only NAPLS-3 data.

**Conclusion:**

The results of this study suggest that a machine learning model trained to predict conversion to psychosis on one dataset can be used to train an independent dataset. Performance on the test set was not in the range necessary for clinical application, however. Possible reasons that limited performance are discussed.

## Background

In a prior priority data letter in *The American Journal of Psychiatry*, we reported that machine learning could be used to predict conversion to psychosis in individuals at clinical high risk (CHR) for psychosis with up to 90% accuracy using the North American Prodrome Longitudinal Study-3 (NAPLS-3) dataset (1). As argued by Cannon (2), a definitive test of our predictive model that was trained on the NAPLS-3 data requires further support through implementation in an independent dataset. In this report, in collaboration with the primary investigators of the NAPLS consortium we tested for model generalization using the previous iteration of NAPLS-3, the NAPLS-2, using the identical machine learning algorithms employed in our previous study. We used the NAPLS-2 dataset as a test set because it used mostly the same measures that were used in the NAPLS-3 to predict conversion to psychosis in a similarly-aged sample.

## Materials and methods

### Participants

NAPLS-2 and 3 are NIMH-funded studies conducted at 8 and 9 sites, respectively. All participants provided written informed consent, including parental consent for minors. The study was approved by all sites’ Institutional Review Boards.

Detailed descriptions of NAPLS-2 and NAPLS-3 participants, including exclusion criteria, are provided in Addington et al. (3) and Addington et al. (4), respectively. Participants were between 12 and 30 years old and were followed up to 2 years. Predictors included those used by the NAPLS-2 calculator (riskcalc.org/napls, see **Table 1**).

**Table 1:** Demographic and clinical information, excluding participants with missing data (*N* = 64).

	NAPLS3 Mean	SD	NAPLS2 Mean	SD	NAPLS2 vs. NAPLS3 t or χ^2^	*p*
*N*	598	–	596	–	–	–
Age in Years*	18.79	4.03	18.51	4.27	-1.16	.25
*N* With First Degree Relative with Psychosis*	117	–	96	–	2.44	.12
*N* Without First Degree Relative with Psychosis*	481		500			
BACS Symbol Coding Raw Score*	54.74	13.21	56.80	13.04	2.72	.007
HVLT Raw Score*	26.39	5.21	25.61	5.15	-2.60	.009
Number of Trauma Types*	1.87	1.61	2.08	1.71	2.20	.028
Decrease in Global Social Functioning Score Over the Past Year*	1.03	.97	.74	1.04	-4.98	<.001
Number of Undesirable Life Events*	9.56	4.86	10.47	5.43	3.06	.002
Rescaled** SIPS Delusions + Suspicions*	3.10	1.46	2.61	1.57	-5.60	<.001
SIPS Delusions + Suspicions*	6.80	1.87	6.08	2.23	-6.00	<.001
*N* Converters	62	–	84	–	3.86	.049
*N* Non-Converters	536		512			
Days from Baseline to Conversion	278.0	286.1	219.7	173.4	-1.42	.16

Numbers in parentheses represent the standard deviation unless otherwise stated. BACS, Brief Assessment of Cognition in Schizophrenia; HC, Healthy Control; HVLT, Hopkins Verbal Learning Test; SIPS, Structured Interview for Psychosis-risk Syndromes. *Included as features in machine learning models. **Rescaled such that scores 0-2 = 0, 3 = 1, 4 = 2, 5 = 3, and 6 = 4.

Consistent with prior work (5), conversion to psychosis for a CHR individual was defined as meeting the Presence of Psychotic Symptoms criteria: One of the five Scale of Psychosis-Risk Symptoms (SOPS) positive symptoms must reach a psychotic level of intensity (rated 6) for ≥ 1 hour per day for 4 days per week during the past month, and/or these symptoms seriously impact their functioning. Machine learning models were tested with and without SOPS rescaling prior to analysis [see **Table 1** legend for rescaling procedure, based on Cannon et al. (6)].

### Analyses

Standard machine learning algorithms were employed using Weka software (University of Waikato, New Zealand) and included logistic regression, naive Bayes, a 3 kernel support vector machine, KStar, J48 decision tree, random forest (max depth 2), decision stump (with 100 iterations of AdaBoost), and multilayer perceptron (see explanation of algorithms below). Classifiers were trained using NAPLS-3 data and tested on NAPLS-2 data. Individuals with missing data were excluded from the analyses. Because of class imbalance, prior to machine learning training data for the minority (converter) class was 800% upsampled using the Synthetic Minority Oversampling Technique (SMOTE) (7) to match the majority (nonconverter) class. By providing more training data of the minority class, this procedure helps to refine the machine learning model decision binary and improve performance (7). SMOTE *k* (*n* nearest neighbors) was set to 5.

### Naive Bayes

Naive Bayes (8) compares the probability of observing an converter/non-converter for each test data point according to the equation *P* (*C_i_* | *x*) = (*P* (*C_i_*) * *p* (*x* | *C*_i_)/*p* (*x*) where *P*(*C_i_*) is the prior probability of class *C_i_* (e.g., converter) occurring, *p* (*x* | *C*_i_) is the conditional probability that class *C_i_* is associated with feature observation *x*, and *p* (*x*) is the marginal probability that observation *x* is observed (effectively constant for any given dataset). The joint model (combining all features) can then be expressed as the product of the probabilities for all features, and the algorithm classifies unseen data as converter or non-converter based on the highest probability.

### Support vector machine

SVM classifiers find the maximum-margin hyperplane using only those data instances closest to the separation boundary (i.e. “support vectors”) to determine classification boundaries (9). Both linear and non-linear (using a kernel) classifications can be performed. Polykernel SVM classifiers were evaluated starting with an exponent of 1 and increasing in size until average accuracy (over all 1000 allocations of test/training data) plateaued.

### KStar (K-nearest neighbor)

The K* algorithm operates by assigning new data instances to the class that occurs most frequently amongst the *k*-nearest data points, *y_j_*, where *j* = 1,2…*k* (10). Distance is then used to retrieve the most similar instances from the data set. The K* function is operationalized as K* (*y_i_*,*x*)= -ln P*(*y_i_*,*x*) where P* is the probability of all transformational paths from instance *x* to *y*, i.e., the probability *x* will arrive at *y* via a random walk in feature space.

### AdaBoost

AdaBoost operates by creating multiple weak classifiers that are weighed by their effectiveness at classifying data (11). Initially, a classifier is created with all instances weighted equally. Next, the weights of the incorrectly predicted instances are increased. The instances that are still misclassified are then selected and their weights increased as well, and so forth. After the complete classifier is constructed, each weak classifier then casts a weighted “vote” as to the class membership of each set of individual test data to make a classification decision.

### J48 decision tree

Decision tree classifiers operate hierarchically, with each level representing a feature (e.g., age) (12, 13). Based on the value of that feature the tree either classifies immediately or passes the information to the next level of the tree. The C4.5 algorithm (12) was used for the J48 decision tree, which uses a measure called “information gain” to select each attribute at each stage. In essence, the J48 tree first chooses the feature that most effectively splits the training data into one class or another using a measure called “information gain” (essentially, the effectiveness of feature at classifying data). After this split, the tree then chooses the next most effective feature to split each resulting partition. The process then iteratively repeats until all training data is classified. Performance of the resulting tree is then evaluated on test data.

### Random forest

A random forest is a group of decision trees made up of random partitions of training data (14). Each tree casts a “vote” as to the classification of a testing instance and votes are counted to produce the final classification.

## Results

Demographic and clinical information for and comparisons between NAPLS-2 and NAPLS-3 participants are provided in **Table 1**. NAPLS-2 and -3 individuals significantly differed on most features used in machine learning models. Examining conversion rates, 84 out of the 596 CHR individuals in NAPLS-2 (14%) and 62 out of the 598 CHR participants in NAPLS-3 (10%) with no missing data converted over the course of the follow-up period.

Machine learning performance metrics for each machine learning algorithm are provided in **Table 2**. Briefly, all models performed above chance as evidenced by concordance values [receiver operating characteristic area under the curve (ROC AUC)] values over 50%. Naive Bayes and random forest showed the best overall performance (AUC). Sensitivity and negative predictive values were relatively high and specificity and positive predictive values were relatively low for all models, however. Importantly, overall performance did not match those previously observed when using only NAPLS-3 data (performance metrics from the previous study are also provided in **Table 2**). For example, when exclusively using NAPLS-3 data (including non-rescaled SIPS scores) for training and testing, the most accurate method (random forest) showed 90% accuracy with 79% sensitivity and 96% specificity (1). When a NAPLS-3-based random forest model was tested on NAPLS-2 data, however, accuracy, sensitivity, and specificity were reduced to 77%, 41%, and 83% (respectively) (**Table 2**, bottom half).

**Table 2:** Predicting conversion to psychosis using NAPLS-3 clinical/demographic data for training and NAPLS-2 data for testing using various machine learning methods.

Method	Acc		Sn		Sp		PPV		NPV		ROC AUC	
SIPS Rescaled												
Decision Stump with AdaBoost	79		33		87		29		89		66	
J48 Decision Tree	82		10		94		21		86		56	
KStar	72		20		81		15		86		52	
Logistic Regression	75		51		79		29		91		67	
MLP*	70		36		76		20		88		60	
Naive Bayes	75		58		78		30		92		70	
Random Forest	78		38		85		29		89		70	
SVM (3 Kernel)	70		36		76		20		88		56	
SIPS Non-Rescaled	NAPLS-3 Train, NAPLS-2 Test						*NAPLS-3 Train, NAPLS-3 Test*					
Decision Stump with AdaBoost	79	***71***	31	***43***	87	***88***	27	***68***	88	***73***	68	***74***
J48 Decision Tree	81	***85***	20	***76***	91	***90***	28	***82***	88	***87***	55	***86***
KStar	70	***78***	21	***77***	78	***79***	14	***69***	86	***86***	57	***84***
Logistic Regression	72	***68***	51	***35***	75	***87***	25	***61***	90	***70***	66	***68***
MLP*	66	***73***	57	***48***	67	***88***	22	***70***	91	***75***	64	***77***
Naive Bayes	66	***70***	55	***45***	68	***84***	22	***62***	90	***73***	65	***68***
Random Forest	77	***90***	41	***79***	83	***96***	28	***92***	89	***89***	69	***96***
SVM (3 Kernel)	69	***74***	45	***41***	73	***93***	22	***78***	89	***73***	59	***67***

Performance from our previous study (1) using NAPLS-3 data (only) for training and testing are provided in shaded bold italic to the right of each metric for comparison (SIPS were non-rescaled only for the previous analysis). Values are percentages. Acc, Accuracy; NPV, Negative Predictive Value; PPV, Positive Predictive Value; ROC AUC, Receiver Operating Characteristics Area Under the Curve; Sn, Sensitivity; Sp, Specificity. *Multilayer perceptron (MLP) with 2 hidden layers (5 nodes in the first and 2 in the second).

## Discussion

The results of this study suggest that a machine learning model trained to predict conversion to psychosis on one dataset can be used to train an independent dataset. In this analysis, however, we did not achieve the high level of accuracy as in the training sample and classification success was not in the range generally considered necessary for clinical application (80% positive and negative predictive value). Notably most models in this generalization analysis struggled to identify positive cases (converters), the minority outcome in both samples, although they did show high specificity and AUC scores that were above chance.

One likely reason for the discrepancy in performance is that models that exclusively use NAPLS-3 data may have overfit to that dataset. Chekroud et al. (15) have recently emphasized that machine learning faces a generalizability issue in outcome prediction in studies of psychiatric disorders in that models often do not perform well when tested on unseen data. Consistent with our findings, recent prior work that also sought to predict conversion to psychosis in CHRs observed a significant reduction in performance (from 85% accuracy on the trained sample to 73% on the independent test sample) when a trained model was tested on an independent dataset (16). Although all algorithms here did perform above chance, the fact that they did not perform as well as a model trained within one dataset as in our previous study is in conceptual agreement with the findings of Chekroud et al. (15). An additional potential reason for poor performance is that the assumption of stationarity, core to machine learning methods including random forest, was not met since the levels of key predictor variables differed significantly across the two samples (17) (as shown in **Table 1**). This presents a significant challenge for researchers seeking to develop generalizable prediction tools using machine learning approaches. One approach to improving generalizability that machine learning researchers are actively investigating is the application of *domain adaptation* methods, to apply in cases in which source and target domains have different distributions but identical underlying predictive features (18). As recently reviewed by Smucny et al. (19), domain adaptation methods have achieved some success when using magnetic resonance imaging data to predict various outcomes, although only a few studies thus far have been conducted and domain adaptation procedures are still being developed and refined. Finally, it should be noted that the power of the model may have been limited by the relatively low sample size of the minority class in the training dataset (*n =* 62). Hence, while we are not “there yet” (1), we believe that the field should eschew nihilism regarding the application of predictive analytics to unique and powerful datasets (such as those collected by the NAPLS consortium) in pursuit of a personalized medicine approach to early intervention for young people with psychosis.

## Data Availability

The data analyzed in this study is subject to the following licenses/restrictions: The NAPLS-2 dataset is only available to NAPLS-2 investigators and their collaborators. Requests to access these datasets should be directed to tyrone.cannon@yale.edu. NAPLS-3 data is available for download on the National Institutes of Mental Health Data Archive.
